# Supplementary material for: Stability of Dengue 2 Nonstructural Glycoprotein 1 (NS1) Is Affected by the Nature of Basic Residue at Position NS1-324
Source: Curr Issues Mol Biol. 2023 Feb 14;45(2):1644–54. doi: 10.3390/cimb45020106 (PMC9955058; doi:10.3390/cimb45020106)
Supplement: Supplementary file 1 [file cimb-45-00106-s001.zip › cimb-2107494-SM.pdf]

current issues in molecular biology

MDPI

Supplementary Materials

Table S1. List of primers for RT-qPCR analysis.

Reference	Gene	Forward Primer	Reverse Primer
NM_002046.5	**GAPDH**	GGGAGCCAAAAGGGTCATCA	TGATGGCATGGACTGTGGTC
NM_000576.2	**IL-1β**	TTCGACACATGGGATAACGA	TCTTTTCAACACGCAGGACAG
NM_000600.3	**IL-6**	AAAGCTGCGCAGAATGAGAT	AAAGAGGCACTGGCAGAAAA
NM_000454.4	**SOD1**	GAAGGTGTGGGGAAGCATTA	ACATTGCCCAAGTCTCCAAC

Table S2. Amino-acid residues at positions NS1-272 and NS1-324 among DENV-2 strains

Place of isolation	Year of isolation	NS1-272	NS1-324	Genbank accession number
Reunion island	2018	K	K	MN27404
Malaysia	2015	K	K	KY923048
Thailand	1997	K	R	U87411
Pakistan	2014	K	R	KF360005
Kenya	2017	K	R	MG779195
United-States	2016	K	R	KX702404
Venezuela	2016	K	R	MH215277
Tanzania	2014	R	R	MG189962
China	2011	R	R	JX470186

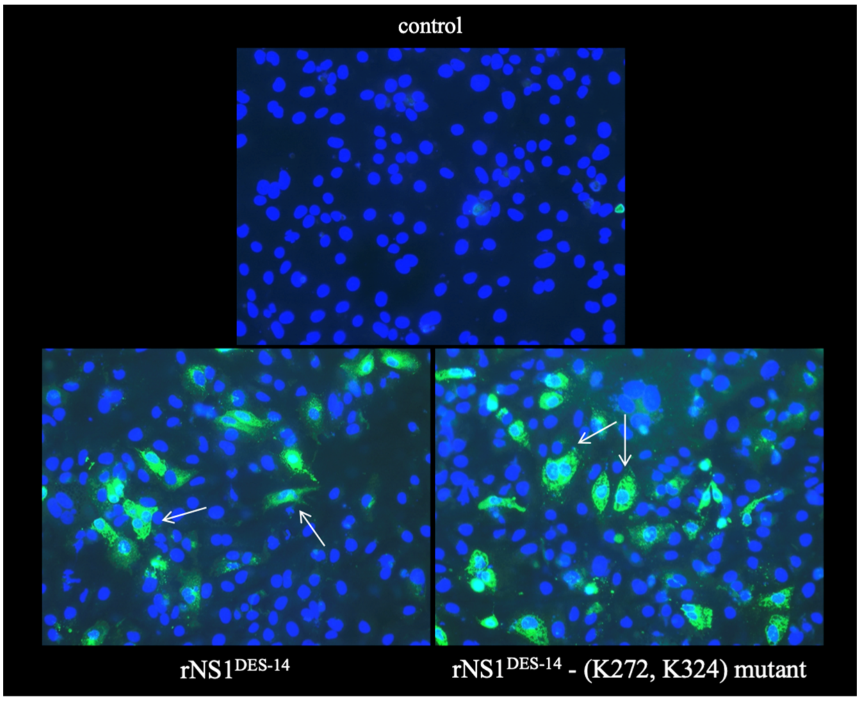

Figure S1. Expression of recombinant DES-14 NS1 proteins in Huh7 cells. Huh7 cells were transfected 24h with pcDNA3 plasmids expressing FLAG-tagged DES-14 rNS1 protein or its mutant or mock-transfected (control). Indirect immunofluorescence assays were performed as previously described [8]. Anti-FLAG antibody (green) served as primary antibody and goat anti-mouse IgG AlexaFluor 488 as secondary antibody [8]. The arrows indicate the NS1-positive cells. The nuclei were stained with DAPI (blue). A Nikon Eclipse E2000-U microscope was used for visualization of the fluorescence. A magnification of x200 was used throughout.
